# Influence of Social Support on Self-Management Behavior of Hemodialysis Patients: Focusing on the Mediating Effect of Self-Esteem

**DOI:** 10.3390/healthcare14162507

**Published:** 2026-08-12

**Authors:** Sung Min Kim, JaeLan Shim

**Affiliations:** College of Nursing, Dongguk University, WISE Campus, 123 Dongdae-ro, Gyeongju 21936, Republic of Korea

**Keywords:** hemodialysis patients, self-esteem, self-management behavior, social support, social cognitive theory

## Abstract

**Highlights:**

**What are the main findings?**

- Perceived social support, self-esteem, and self-management behavior were significantly and positively correlated among patients undergoing hemodialysis. The findings were consistent with partial statistical mediation of self-esteem in the association between perceived social support and self-management behavior.
- Among the sources of perceived social support, friend support had the lowest mean score. Descriptively, perceived social support, self-esteem, and self-management behavior were lowest in the 5–<10-year dialysis group; however, these findings should not be interpreted as evidence of longitudinal change or a distinct vulnerable period.

**What are the implications of the main findings?**

- Nursing interventions may benefit from a dual approach that strengthens self-esteem as an internal psychological resource while reinforcing supportive social resources to promote self-management among patients undergoing hemodialysis.
- Regular psychosocial assessment may help identify patients with limited social support during long-term hemodialysis. Structured peer-support and counseling programs may also help address social isolation and strengthen perceived support.

**Abstract:**

**Background/Objectives:** Patients undergoing hemodialysis experience psychosocial burdens that may hinder self-management. Guided by Bandura’s social cognitive theory, this study examined whether self-esteem statistically mediated the association between perceived social support and self-management behavior. **Methods:** A descriptive cross-sectional study was conducted with 145 patients receiving regular hemodialysis at three medical institutions in South Korea. The mediation model was tested using Hayes’ PROCESS macro Model 4 with 5000 bootstrap samples and 95% bias-corrected confidence intervals, adjusting for relevant covariates. **Results:** Self-management behavior was positively correlated with social support (r = 0.41, *p* < 0.001) and self-esteem (r = 0.52, *p* < 0.001). Social support was also positively correlated with self-esteem (r = 0.67, *p* < 0.001). Social support was associated with self-management both directly and indirectly through self-esteem. The bootstrap confidence interval for the indirect association excluded zero, consistent with partial statistical mediation. The indirect association accounted for approximately 45% of the total association. **Conclusions:** Higher perceived social support and self-esteem were associated with better self-management behavior among patients undergoing hemodialysis. Nursing interventions that strengthen supportive social resources and self-esteem may help promote sustained self-management in this population.

## 1. Introduction

Rapid advancements in medical technology have significantly increased life expectancy, leading to a substantial rise in the prevalence of end-stage renal disease (ESRD) globally. Recent estimates indicate that approximately 4.59 million people worldwide were living with kidney failure requiring replacement therapy, including maintenance dialysis or kidney transplantation, in 2023, reflecting a substantial increase in the global burden of advanced kidney disease [1].

Hemodialysis is a life-sustaining treatment for patients with chronic kidney failure, but it also imposes substantial physical, psychological, and social burdens [2,3]. Because patients must undergo treatment several times per week over a prolonged period, they often experience restrictions in daily life, emotional distress, and difficulty sustaining necessary self-management behaviors, including healthy lifestyle practices and physical activity [4].

Specifically, HD patients experience a complex constellation of symptoms—characterized by physical, psychological, and social dimensions—that serve as a primary driver for reduced self-management. The cumulative strain of this multidimensional symptom burden often exceeds the patient’s capacity to maintain necessary health behaviors [5]. Consistent with this, studies have reported that HD patients demonstrate only moderate levels of healthy lifestyle behaviors overall, with physical activity being particularly deficient, suggesting that the demands of HD treatment substantially hinder patients’ ability to engage in health-promoting self-management practices [4,6].

Beyond the burden experienced by patients themselves, HD also creates significant caregiving responsibilities for family members. Research has consistently demonstrated that caregivers of HD patients experience higher levels of burden compared to caregivers of patients who have undergone kidney transplantation [7], and the problems experienced by caregivers whose burden increases complicate patients’ adaptation to treatment [8]. This elevated caregiver burden may in turn indirectly impair patients’ ability to maintain self-management behaviors.

Social support is an important resource for patients undergoing HD because it can reduce stress, facilitate adaptation to chronic illness, and promote coping with treatment-related demands [9,10,11]. Support from family, friends, and healthcare professionals may also help patients maintain self-management behaviors.

Self-management plays a crucial role in enhancing the QOL for patients on HD, encompassing behaviors such as dietary and fluid control, medication adherence, vascular access care, symptom monitoring, and emotional regulation. Self-management plays a crucial role in the care of patients undergoing hemodialysis and includes dietary and fluid control, medication adherence, vascular access care, symptom monitoring, physical activity, and emotional regulation. Effective self-management may contribute to better treatment adherence, improved clinical indicators, and enhanced quality of life [12,13,14].

Recent studies have identified various psychosocial factors associated with self-management among patients undergoing hemodialysis. For example, Park and Jung [14] reported that psychosocial characteristics, including social support and individual psychological factors, were significant correlates of self-management behaviors in patients receiving maintenance hemodialysis.

Among these psychological resources, self-esteem was selected as the potential mediator in the present study because it reflects patients’ overall evaluation of their personal worth and competence. Self-esteem may help patients maintain motivation, cope with chronic illness, and engage in health-promoting behaviors and social relationships [15]. Considering that patients on HD depend on healthcare staff and machinery for survival two to three times a week for prolonged periods [16], they commonly experience helplessness and reduced self-esteem [17]. Therefore, it is vital to support patients on HD in recognizing their worth and fostering confidence, as negative emotions such as fatigue, depression, anxiety, and stress can diminish self-esteem in these patients [18].

**Figure 1:**
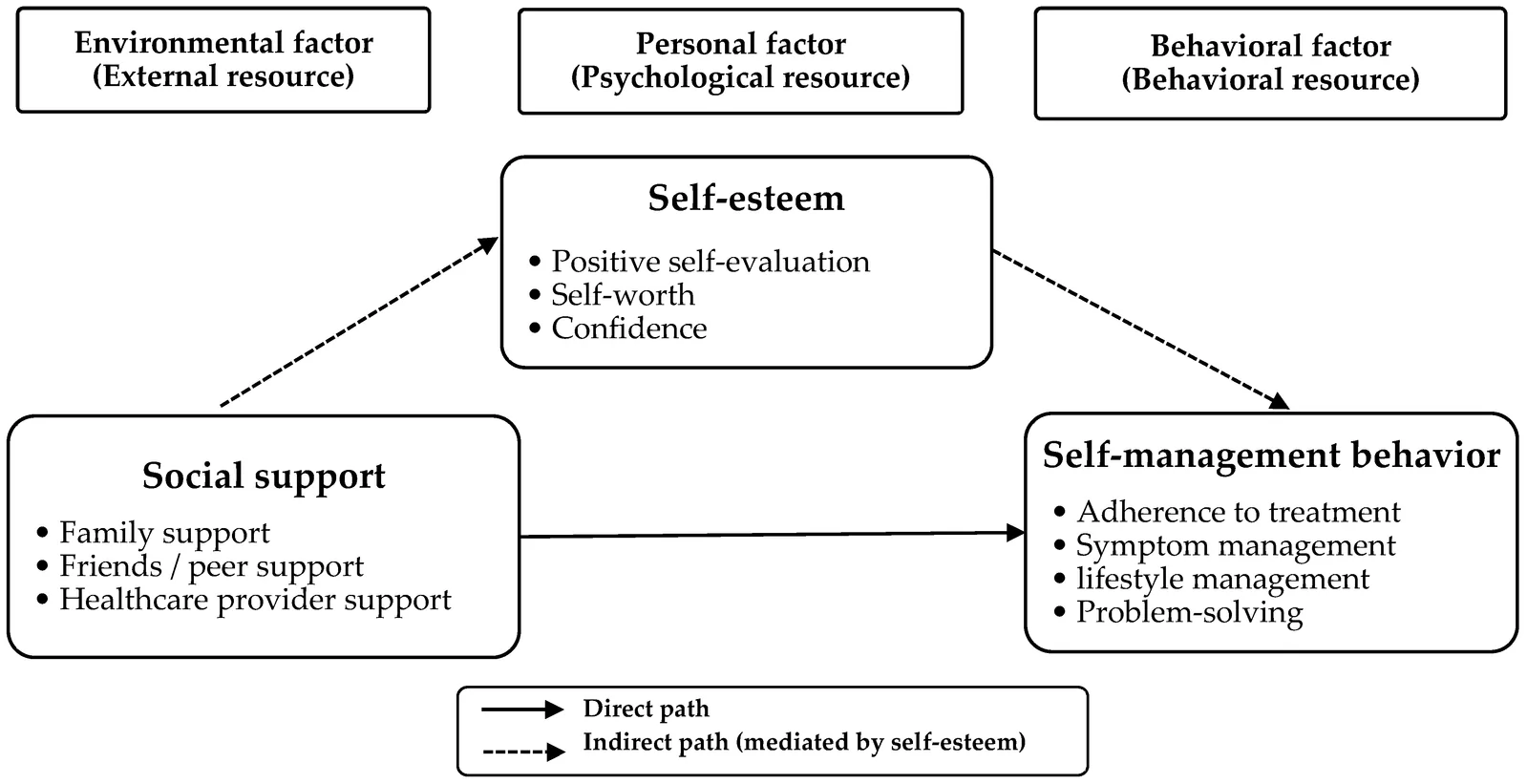
Hypothesized conceptual framework based on Bandura’s Social Cognitive Theory.

Therefore, to explain why self-esteem may serve as the mediating mechanism between social support and self-management, this study draws on Bandura’s social cognitive theory (SCT) [19]. SCT posits that personal factors, behavior, and environmental influences operate as interacting determinants through triadic reciprocal causation [19]. Within this framework, social support represents the environmental factor that provides external resources and reinforcement; self-esteem represents the personal factor that shapes internal self-evaluative processes; and self-management behavior represents the behavioral factor through which patients enact health-promoting actions. The triadic model provides a theoretical basis for hypothesizing that the environmental factor (social support) influences the behavioral factor (self-management) by first altering the personal factor (self-esteem) (Figure 1).

Based on the SCT-guided conceptual framework and previous empirical findings, the following hypotheses were proposed:

**H1.**  *Perceived social support will be positively associated with self-esteem among patients undergoing hemodialysis.*

**H2.**  *Perceived social support will be positively associated with self-management behavior among patients undergoing hemodialysis.*

**H3.**  *Self-esteem will be positively associated with self-management behavior after accounting for perceived social support and relevant covariates.*

**H4.**  *Self-esteem will statistically mediate the association between perceived social support and self-management behavior.*

## 2. Materials and Methods

### 2.1. Aims

Guided by Bandura’s Social Cognitive Theory, this study aimed to examine whether self-esteem, as a personal psychological factor, statistically mediates the association between social support, as an environmental factor, and self-management behavior among patients with ESRD undergoing hemodialysis. The findings were intended to provide a theoretical basis for nursing interventions that enhance social support and self-esteem to promote self-management behavior.

### 2.2. Design

A descriptive cross-sectional design was employed.

### 2.3. Participants

Participants aged 19 years and older who had been diagnosed with ESRD and were undergoing HD two to three times a week at one university hospital and two dialysis centers in South Korea between October 2022 and January 2023 were recruited. The specific inclusion criteria were as follows: (a) diagnosis of ESRD as evidenced by a glomerular filtration rate (GFR) of <15 mL/min/1.73 m^2^ or kidney injury by a nephrologist; (b) considering that it takes approximately six months until patients on HD are able to psychologically accept HD, participants who have been undergoing dialysis for more than six months were included [20]; (c) participants with no cognitive impairments or psychiatric conditions; and (d) provision of written informed consent.

The exclusion criteria were: (a) hospitalization due to an acute change in disease status during HD; (b) patients undergoing temporary dialysis via a catheter.

The required sample size was estimated a priori using G*Power version 3.1.9.7 for multiple linear regression. Based on a significance level of 0.05, statistical power of 0.85, a conventional medium effect size of f^2^ = 0.15, and 10 predictor parameters in the largest regression equation, the minimum required sample size was 131. The final sample of 145 participants therefore exceeded this requirement. There were no questionnaires with many missing responses or unreliable response patterns. Therefore, no missing-data imputation was required, and a total of 145 participants were included in the analysis.

### 2.4. Instrument

#### 2.4.1. Social Support

Social support was measured using the Korean version of the Multidimensional Scale of Perceived Social Support (MSPSS). Although the original MSPSS consists of three subscales (family, friends, and significant other) [21], the Korean adaptation developed by Shin and Lee [22] replaced the “significant other” subscale with “healthcare professional support” to better reflect the Korean healthcare context and characteristics of patients with chronic illness. Therefore, the present study assessed perceived support from family, friends, and healthcare professionals.

The MSPSS scale consists of 12 items for family support, friend support, and healthcare professional support. Each item is rated on a five-point Likert scale, ranging from 1 (“strongly disagree”) to 5 (“strongly agree”). The reliability (Cronbach’s α) of the tool was 0.85 at the time of development, with 0.85, 0.75, and 0.72 for family support, friend support, and healthcare professional support, respectively. The reliability (Cronbach’s α) of the tool was 0.87 in this study, with 0.94 for both family support and friend support, and 0.89 for healthcare professional support.

#### 2.4.2. Self-Management Behavior

Self-management behaviors among patients on HD were assessed using the self-management behavior scale developed by Song and Lin [23] and adapted by Cha and Kang [24]. This tool consists of 20 items within four domains: problem-solving skills (5 items), self-care (7 items), emotional management (4 items), and partnership (4 items). Each item is rated on a four-point Likert scale from 1 (“never”) to 4 (“always”). A higher score indicates a higher level of self-management. The reliability (Cronbach’s α) of the tool was 0.87 at the time of development and 0.92 in this study.

#### 2.4.3. Self-Esteem

Self-esteem was measured using 25 items translated from the Self-Esteem Inventory by Kang [25,26]. It contains 25 items within four domains: self-depreciation (5 items), social relations (7 items), leadership and popularity (6 items), and self-assertion and anxiety (7 items). Each item is rated on a four-point Likert scale from 1 (“strongly disagree”) to 4 (“strongly agree”). A higher score indicates a higher level of self-esteem. The reliability (Cronbach’s α) of the tool was 0.94 at the time of development and 0.95 in this study.

### 2.5. Validity, Reliability and Rigor

Permission was obtained from the original author of the questionnaire before it was disseminated in the present study. Only instruments in Korean with confirmed reliability and validity were used in the test of the various studies. All instruments used in the study have a high internal consistency.

### 2.6. Ethical Considerations

This study was approved by the Institutional Review Board at the study facility prior to data collection (Approval Code: DGU IRB 2022-0015). Data were collected from patients at one university hospital and two private hospitals with a dialysis center from October 2022 to January 2023 after obtaining written informed consent from the participants.

### 2.7. Data Collection

Data were collected either before dialysis or during dialysis when vital signs had stabilized. After explaining the purpose of this study, a consent form was obtained; it was explained that the subject could stop data collection at any time if they wished. For participants with difficulty completing the questionnaire due to poor vision, advanced age, or other challenges, the researcher provided assistance by reading the questions aloud and recording the participants’ responses.

### 2.8. Data Analysis

The collected data were analyzed using the SPSS/WIN 27.0 software (IBM Corp., Armonk, NY, USA) and SPSS PROCESS macro (Version 4.2) [27]. Participants’ general characteristics and measurement variables were analyzed using descriptive statistics and included as covariates in the mediation analysis to reduce potential confounding.

Differences in social support, self-management behaviors, and self-esteem according to general characteristics were analyzed using an independent t-test, a one-way ANOVA, and Scheffé’s test. The relationship between social support, self-management behaviors, and self-esteem among the participants was analyzed with Pearson correlation, and the mediating effect of self-esteem in the relationship between social support and self-management behaviors was analyzed using the SPSS PROCESS Macro model 4 proposed by Hayes [25]. The statistical significance of the mediating effect was tested through bootstrapping at 5000 iterations with a 95% confidence interval (CI).

## 3. Results

### 3.1. Differences in Self-Management Behavior According to Patients’ General and Disease-Related Characteristics

Table 1 shows the differences in self-management behavior according to patients’ general and disease-related characteristics.

Participants had a mean age of 62.97 years (SD 10.45). Self-management behavior differed significantly according to marital status, main caregiver, insurance type, kidney transplant waiting status, hemodialysis education experience, and subjective health status (Table 1). Higher self-management scores were observed among married participants, those with a main caregiver, those covered by national health insurance, those on the transplant waiting list, those who had received hemodialysis education more than once, and those who perceived their health as good. No significant differences were found according to age, gender, religion, current job, or hemodialysis duration.

### 3.2. Levels of Social Support, Self-Management Behavior, and Self-Esteem Among Participants

Table 2 shows the levels of social support, self-management behavior, and self-esteem.

**Figure 2:**
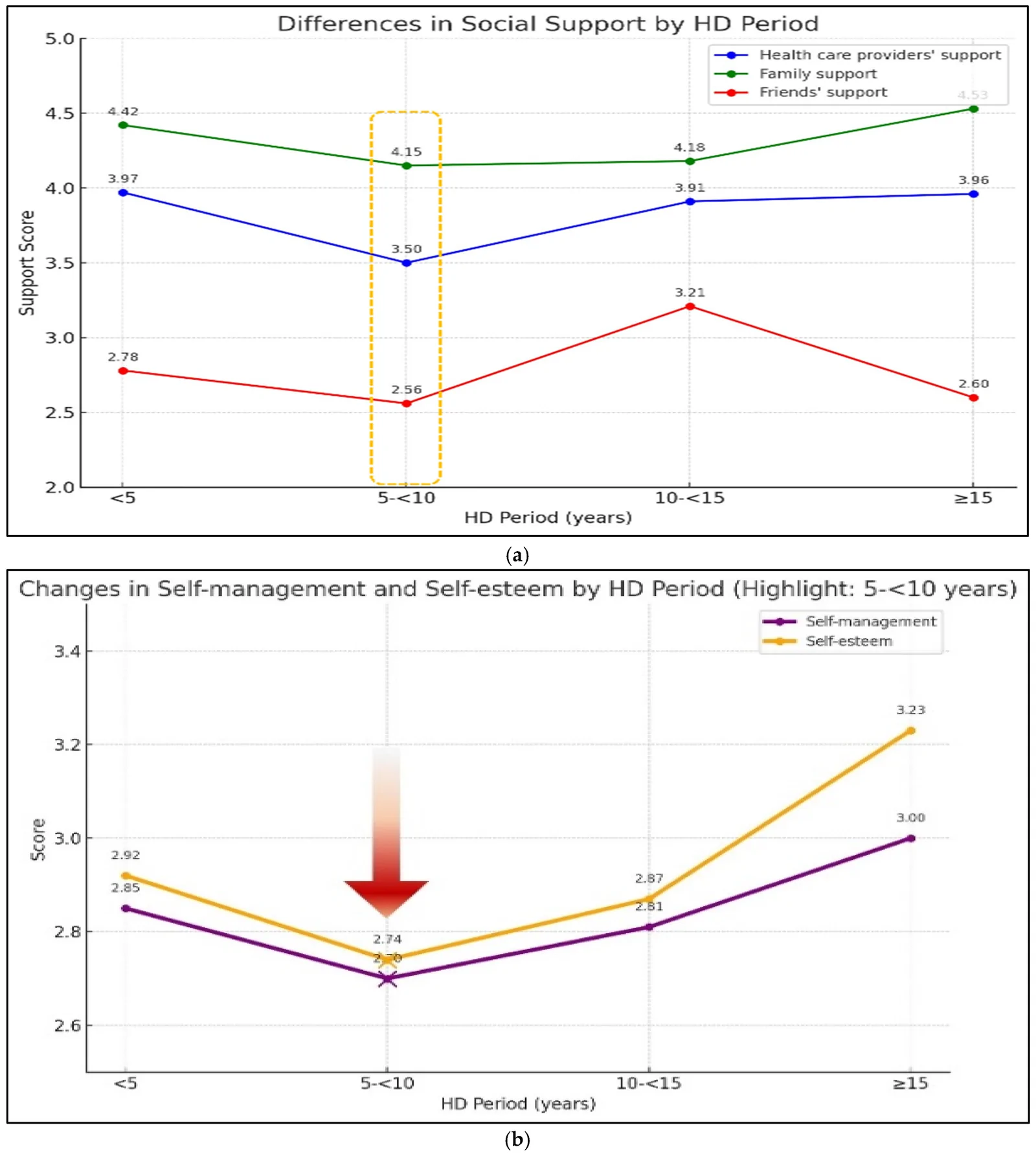
Differences in psychosocial variables according to dialysis duration: (**a**) perceived social support; (**b**) self-management behavior and self-esteem.

Mean scores were 3.62 (SD 0.71) for social support, 2.82 (SD 0.52) for self-management behavior, and 2.90 (SD 0.56) for self-esteem (Table 2). Within social support, family support was highest, and friends’ support was lowest. Within self-management behavior, fluid and weight control showed the highest mean score, whereas self-advocacy and emotion control showed the lowest. Descriptively, the 5–<10-year dialysis group had the lowest mean scores for perceived social support, self-management behavior, and self-esteem (Figure 2).

### 3.3. Association Among Social Support, Self-Management Behavior, and Self-Esteem

Table 3 shows the correlations among the measurement variables. Social support was positively correlated with both self-esteem (r = 0.67, *p* < 0.001) and self-management behavior (r = 0.41, *p* < 0.001), and self-esteem was also positively correlated with self-management behavior (r = 0.52, *p* < 0.001) (Table 3).

### 3.4. Mediating Effect of Self-Esteem in the Relationship Between the Participant’s Social Support and Self-Management Behavior

**Figure 3:**
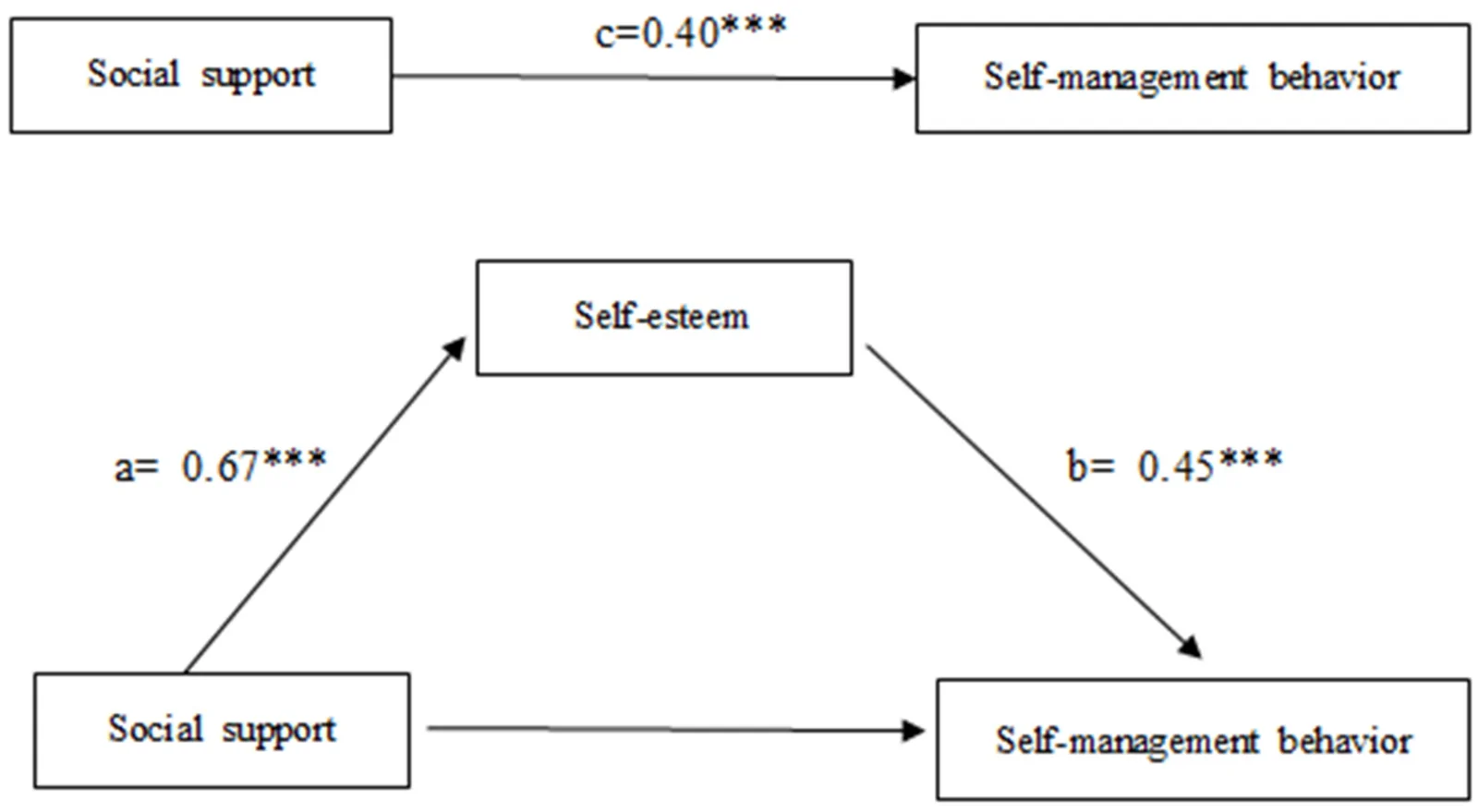
Mediating effect of self-esteem in the relationship between social support and self-management behavior, based on Bandura’s social cognitive theory. Note. *** *p* < 0.001.

This study examined the mediating role of self-esteem in the relationship between social support and self-management behaviors in HD patients (Table 4). Control variables related to self-management behaviors in HD patients, such as marital status, main caregiver, health insurance type, Kidney transplant waiting status, previous HD education, and health status, were included in the statistical analysis. The results showed that social support predicted self-esteem (a = 0.67, SE = 0.05, *p* < 0.001). Self-esteem significantly predicted self-management behaviors (b = 0.45, SE = 0.09, *p* < 0.001). Social support had a direct effect on self-management behaviors (c′ = 0.16, SE = 0.07, *p* = 0.026). Bootstrap analysis using bias-corrected percentiles showed that the 95% confidence interval did not include zero, confirming that self-esteem partially mediated the association between social support and self-management behaviors. This mediating effect explained 45% of the total effect (Table 4) (Figure 3).

## 4. Discussion

This study aimed to verify the mediating effect of self-esteem, an internal psychological factor, on the relationship between social support based on Bandura’s social cognitive theory (SCT) and self-management behaviors, which are critical for hemodialysis patients to develop a positive perception of their disease and to accept it emotionally. Given the prolonged and complex nature of the hemodialysis treatment process that patients must navigate independently, self-esteem serves as a fundamental psychological resource for enhancing self-management capacity. To our knowledge, this study provides meaningful evidence highlighting the importance of strengthening self-esteem as a key factor in enabling patients to better engage with and manage their own health challenges.

While direct comparisons of results are challenging due to the lack of prior studies specifically examining the mediating effects among patients on HD, a study exploring a predictive model of health-promotion behavior in patients on HD [28,29,30] found that they are able to develop confidence and a positive perception of their actions through the mediation of self-esteem and self-efficacy, thereby aiding their health promotion behaviors. This is similar to the results of the present study.

The mean self-esteem score in this study was 2.90 out of 4, which was higher than that reported in a Turkish sample of HD patients [18] but comparable to that in a Brazilian HD population [31]. Several evidence-based factors may account for these score differences. Sociocultural context plays a significant role, as cultural norms surrounding illness, social roles, and family support structures differ substantially across populations [30,32,33]. Among Korean HD patients specifically, uncertainty, economic status, and employment have been identified as the strongest predictors of self-esteem, collectively explaining 30% of its variance [33], suggesting that differences in participants’ socioeconomic profiles and disease-related uncertainty across studies may substantially contribute to the observed score variations. Additionally, differences in the measurement instruments used across studies (e.g., Rosenberg Self-Esteem Scale vs. Coopersmith Self-Esteem Inventory) limit the direct comparability of self-esteem scores.

In patients undergoing HD, high self-esteem not only fosters voluntary participation in their treatment [32], but also enhances their capacity for healthy social relationships, contributes to positive health outcomes, and acts as a driving force for maintaining their own life [15], effectively contributing to facilitating health-promotion behaviors and disease management among patients on HD [17].

Patients on HD rely on the dialysis machine to sustain life until they can receive a kidney transplant or until death. The prolonged duration of illness triggers depression and helplessness, which in turn diminishes self-esteem [34]. The results of this study shed light on a generally negative emotional state among patients on HD, as evidenced by 70.4% of the participants choosing “average or unhealthy” in their self-rated health, thus highlighting the need for proactive strategies to increase self-esteem among patients with chronic conditions. Thus, future studies should first explore the predictors of self-esteem in patients on HD and subsequently develop and evaluate interventions that boost self-esteem in this patient population.

These findings are consistent with the possibility that self-esteem may explain part of the association between social support and self-management. In addition, our findings complement those of Parviniannasab et al. [9], who reported that hope mediated the relationships among uncertainty, social support, and self-management. Whereas their study highlighted hope as a future-oriented psychological resource, the present study suggests that self-esteem, reflecting individuals’ global sense of self-worth and competence, may represent another important psychological mechanism associated with self-management. Taken together, these findings indicate that multiple internal psychological resources may help explain how psychosocial environments are associated with behavioral adaptation in patients undergoing hemodialysis. These findings extend recent evidence demonstrating that psychosocial factors play an important role in self-management among patients undergoing hemodialysis [14].

Also, the indirect association through self-esteem offers a theoretically grounded interpretation of the mediation result. Within the SCT framework, supportive interactions may function as environmental resources by providing encouragement, feedback, practical assistance, and social reinforcement. These resources may be associated with a more positive evaluation of personal worth and competence, which may, in turn, be associated with greater persistence in demanding self-management activities (Figure 1).

The present findings may also be understood within the broader literature on internal psychological processes underlying adaptation-related outcomes. Di Gesto et al. [35] found that psychological inflexibility statistically mediated the association between emotion-regulation difficulties and perceived stress among ICU nurses. Although their study involved a different population and outcome, it provides conceptual evidence that internal psychological processes may help explain associations between psychosocial factors and health-related outcomes across distinct healthcare contexts. Similarly, the present findings suggest that self-esteem may represent a personal psychological factor statistically linking perceived social support with self-management behavior among patients undergoing hemodialysis.

The findings suggest that psychosocial adaptation to hemodialysis is a dynamic process that changes throughout long-term treatment rather than remaining stable over time. Although patients may gradually develop greater confidence and self-management skills, sustaining adequate social support may become increasingly difficult. Because social support is closely associated with treatment adaptation, quality of life, and overall health outcomes among patients receiving dialysis [9,35], regular assessment of psychosocial needs and individualized supportive interventions should be incorporated into long-term nursing care. Future longitudinal studies are needed to identify factors contributing to changes in social support across different stages of hemodialysis.

In particular, our results indicated that support from friends was lower than that from healthcare professionals or family. A study examining the relationships between self-efficacy, social support, and family resilience among 183 patients at a hemodialysis center in Iran and their 183 family members also found that support from friends was the lowest among social forms of support, which is similar to the results of this study [36]. This finding may reflect the restricted social networks experienced by patients undergoing long-term hemodialysis. Comparisons of the family and friend subscales may remain informative; however, comparisons involving the total score or the relative ranking of all three support sources should be interpreted cautiously because the Korean version of the MSPSS used in the present study replaces the original “significant other” subscale with healthcare professional support.

Given the risk of social isolation associated with long-term, repetitive dialysis schedules, the low level of friend support observed in this study suggests the need for structured support measures beyond general encouragement, including peer-support groups, hospital-based social welfare counseling, and psychological support services. The demanding nature of the treatment regimen, coupled with its accompanying side effects-such as fatigue-can hinder the development and maintenance of social relationships beyond close family members [35]. So further research is needed to explore why HD patients have low support from their peers and develop programs or nursing interventions to improve this. In addition, these results can also be interpreted in relation to patients’ help-seeking behaviors. Even if potential support resources exist, it may depend on the patient’s awareness of such support and their willingness and ability to request help regarding symptoms in a timely manner [37]. Since this study did not directly evaluate help-seeking behaviors regarding support, it is thought that future research would be useful to investigate the barriers to help-seeking behaviors and symptom disclosure in the hemodialysis patient population using hemodialysis-specific assessment tools [38].

Future longitudinal and intervention studies are warranted to clarify how social support and self-esteem interact over time to influence self-management behaviors. Particular attention should be given to identifying patients who become psychosocially vulnerable during long-term hemodialysis and to developing theory-based nursing interventions that strengthen both external social resources and internal psychological resources. Such efforts may help sustain self-management behaviors and improve long-term health outcomes among patients undergoing hemodialysis.

In this study, it was confirmed that the support score from healthcare providers among social support was the second lowest after support from friends. Healthcare providers who regularly see dialysis patients play a critical role in assessing patient adherence and suggesting strategies to improve adherence [39], and healthcare providers’ support was a significant predictor of patient health outcomes [38]. A partnership between healthcare providers and patients based on knowledge sharing and recognition of the value of patients’ knowledge and experience in self-management of patients with chronic diseases remains a core principle of patient-centered care [13]. However, these findings should also be interpreted within the broader context of symptom management in hemodialysis. Beyond limitations in interpersonal support, previous qualitative evidence suggests that systemic inefficiencies and chronic self-regulatory burden may further hinder patients’ ability to sustain effective symptom management and self-management over time [36].

Although these factors were not directly measured in the present study, considering them may help provide a more comprehensive understanding of the challenges faced by this population. Future research should examine these structural and psychological barriers more explicitly to support more holistic and tailored care.

A key significance of this study is that it confirmed the association between social support and self-management behaviors and shed light on the importance of self-esteem as a psychological factor associated with self-management behaviors in patients on HD who develop several complications due to the lengthy duration of illness. In relation to psychosocial interventions, the role of the healthcare professional is to encourage the patient to accept the limitations caused by the illness based on self-esteem, practice self-management, assess the patient’s needs, identify potential problems, and gather information for treatment planning.

### Limitations

First, this study employed a cross-sectional design, which precludes establishing temporal precedence among social support, self-esteem, intrinsic motivation, and self-management. Consequently, the observed mediating relationships should be interpreted as statistical associations rather than evidence of causal pathways. Future longitudinal or experimental studies are needed to clarify the temporal sequence and verify the proposed mediation model. Second, the study relied exclusively on self-reported measures of social support, self-esteem, and self-management behavior, without including objective clinical indicators to validate patients’ reported behaviors.

Future research requires studies that verify self-management behaviors using objective indicators such as mortality rates, rehospitalization rates, or clinical parameters.

Third, although self-esteem was examined as a mediator, other potentially important factors that could influence the relationships between study subjects, such as underlying psychological state, daily family support, and disease severity, were not controlled. Therefore, the findings should be interpreted with caution. In the future, longitudinal studies are required to identify long-term and causal changes among variables over time using larger and more diverse samples.

Fourth, this study did not consider treatment-related clinical variables specific to hemodialysis, such as dialysis adequacy, duration of dialysis, type of vascular access, and comorbidities. All of these variables can independently influence patients’ self-management behaviors. Future research should include dialysis adequacy indicators, vascular access characteristics, and comorbidity burden as covariates to provide a more complete and clinically accurate understanding of the determinants of self-management behaviors in hemodialysis patients.

Another limitation is that several clinically relevant factors that may influence self-management among patients undergoing hemodialysis, including comorbidity burden, dialysis adequacy, symptom severity, cognitive function, depression and anxiety status, and history of treatment adherence, were not included. Therefore, future studies should consider incorporating these additional variables to provide a more comprehensive understanding of factors associated with self-management in this population.

## 5. Conclusions

Patients with end-stage renal failure who undergo hemodialysis depend on long-term dialysis treatment due to the chronic and complex nature of their disease, and must manage their chronic disease while simultaneously maintaining social roles and responsibilities along with continuous dialysis and outpatient treatment. In such a situation, self-esteem serves as an important psychosocial resource to effectively perform the patient role.

In essence, the observed associations were consistent with a partial mediating role of self-esteem in the relationship between perceived social support and self-management behavior among patients undergoing hemodialysis.

Chronic diseases suffered by patients on HD cannot be resolved through short-term treatment and instead require extensive physical and mental health management and self-care [40]. In addition, readmission following discharge may take a toll on patients’ QOL due to financial constraints and poor physical activity [6]. Therefore, self-esteem as well as social support should essentially be enhanced to foster self-management behaviors among patients, which requires a multidisciplinary effort involving physicians, nurses, and nutritionists; from an organizational perspective, it is essential to build a practical and supportive infrastructure to sustain these health-promoting behaviors over the long term.

### Implications for Nursing Practice

The present findings also have practical implications for nursing practice. Rather than focusing solely on disease management, nursing interventions should incorporate psychosocial strategies that enhance patients’ psychological resources and coping capacity. Evidence-based approaches such as nurse-led self-management education, individualized goal setting, motivational interviewing, peer-support programs, and strengths-based counseling may help foster self-esteem, patient empowerment, and adaptive coping. Furthermore, encouraging family involvement and supportive interactions with healthcare professionals may strengthen perceived social support, which could contribute to improved self-management among patients undergoing hemodialysis.

In this study, participants who had undergone hemodialysis for 5–10 years reported lower levels of perceived social support, self-esteem, and self-management than those in other dialysis-duration groups. Although these findings may suggest that psychosocial adaptation differs according to dialysis duration, the present analyses were descriptive and were not designed to determine whether the 5–10-year period represents a particularly vulnerable stage. Accordingly, nursing interventions aimed at maintaining social support and enhancing self-esteem may be particularly important during this stage.

In addition, this observation should be interpreted with caution and suggests that research is needed to clarify whether psychosocial needs change over the course of long-term hemodialysis.

## Figures and Tables

**Table 1:** Differences in self-management behavior according to patients’ general and disease-related characteristics (N = 145).

Characteristics	Category	n (%) or M (SD)	Self-Management Behavior		
			M (SD)	t or F (*p*)	Scheffé Test *
Age(years)	≤49	16 (11.0)	2.64 (0.52)	0.90 (0.442)	
	50–64	59 (40.7)	2.88 (0.50)		
	65–74	50 (34.5)	2.80 (0.54)		
	≥75	20 (13.8)	2.83 (0.55)		
		62.97 (10.45)			
Gender	Male	105 (72.4)	2.79 (0.53)	−0.93 (0.353)	
	Female	40 (27.6)	2.88 (0.49)		
Marital status	Married	125 (86.2)	2.86 (0.50)	**2.38 (** **0** **.019)**	
	Unmarried	20 (13.8)	2.56 (0.58)		
Religion	Yes	92 (63.4)	2.86 (0.51)	1.19 (0.235)	
Current job	Yes	52 (35.9)	2.83 (0.50)	0.17 (0.864)	
Main caregiver	Present	119 (82.1)	2.89 (0.51)	**3.93 (<** **0** **.001)**	
	Absent	26 (17.9)	2.47 (0.46)		
Type of insurance	NHI	100 (69.0)	2.92 (0.51)	**3.60 (<** **0** **.001)**	
	Medical aid	45 (31.0)	2.59 (0.48)		
HD period(years)	<5	80 (55.2)	2.86 (0.53)	2.04 (0.111)	
	5–<10	35 (24.1)	2.64 (0.47)		
	10–<15	17 (11.7)	2.81 (0.58)		
	≥15	13 (9.0)	3.00 (0.50)		
		6.73 (6.90)			
KTPL waiting status	Yes	49 (33.8)	2.94 (0.47)	**2.02 (** **0** **.046)**	
Hemodialysis education experience	No ^a^	33 (22.8)	2.62 (0.57)	**4.88 (** **0** **.009)**	a < c *
	One time ^b^	57 (39.3)	2.78 (0.47)		
	More than two times ^c^	55 (37.9)	2.96 (0.51)		
Subjective health status	Good ^a^	43 (29.6)	3.06 (0.52)	**9.04 (<** **0** **.001)**	b, c < a *
	Moderate ^b^	49 (33.8)	2.80 (0.45)		
	Bad ^c^	53 (36.6)	2.63 (0.51)		

Abbreviations: HD, hemodialysis; KTPL, kidney transplantation; M, mean; SD, standard deviation; NHI, National Health Insurance. Note: * Post hoc test was calculated using Scheffé’s method.

**Table 2:** Descriptive statistics for social support, self-management behavior, and self-esteem.

Variables	Subdomain	Mean (SD)	Range
Social support		**3.62 (0.71)**	1∼5
	Family support	4.31 (0.97)	
	Healthcare providers’ support	3.83 (0.81)	
	Friends’ support	2.73 (1.19)	
Self-management behavior		**2.82 (0.52)**	1∼4
	Problem solving and communication	2.79 (0.63)	
	Fluid and weight control	3.40 (0.52)	
	Diet and hemodialysis	2.92 (0.53)	
	Self-advocacy and emotion control	2.49 (0.64)	
Self-esteem		**2.90 (0.56)**	1∼4
	Self-deprecation	2.80 (0.63)	
	Relationship with others	3.26 (0.67)	
	Leadership and popularity	2.70 (0.57)	
	Self-assertion and anxiety	2.79 (0.62)	

Abbreviation: SD, standard deviation.

**Table 3:** Correlations between social support, self-management behavior, and self-esteem.

Variables	Social Support	Self-Esteem	Self-Management Behavior
	r (*p*)	r (*p*)	r (*p*)
Social support	1		
Self-esteem	0.67 (<0.001)	1	
Self-management behavior	0.41 (<0.001)	0.52 (<0.001)	1

**Table 4:** Mediating effect of self-esteem on the relationship between social support and self-management behavior.

Effect	Path	β	SE	t	*p*	95% CI	
						LLCI	ULCI
Direct effect	Social support →Self-management behavior	0.16 (c′)	0.07	2.28	0.026	0.02	0.29
	Social support →Self-esteem	0.67 (a)	0.05	10.70	<0.001	0.53	0.78
Indirect effect	Self-esteem →Self-management behavior	0.45 (b)	0.09	4.66	<0.001	0.24	0.59
Total effect	Social support →Self-management behavior	0.40 (c)	0.06	4.42	<0.001	0.22	0.52

Abbreviations: CI, confidence interval; LLCI, lower limit confidence interval; SE, standard error; ULCI, upper limit confidence interval. a, effect of social support on self-esteem; b, effect of self-esteem on self-management behavior controlling for social support; c, total effect of social support on self-management behavior; c′, direct effect of social support on self-management behavior controlling for self-esteem.

## Data Availability

The original contributions presented in this study are included in the article. Further inquiries can be directed to the corresponding author.

## Abbreviations

The following abbreviations are used in this manuscript:

CI	confidence interval
ESRD	end-stage renal disease
GFR	glomerular filtration rate
HD	hemodialysis
IRB	Institutional Review Board
KTPL	kidney transplantation
LLCI	lower limit of the confidence interval
MSPSS	Multidimensional Scale of Perceived Social Support
NHI	National Health Insurance
QOL	quality of life
SCT	social cognitive theory
SD	standard deviation
SE	standard error
ULCI	upper limit of the confidence interval
